# Supplementary material for: Iterative improvement in the automatic modular design of robot swarms
Source: PeerJ Comput Sci. 2020 Dec 7;6:e322. doi: 10.7717/peerj-cs.322 (PMC7924708; doi:10.7717/peerj-cs.322)
Supplement: Supplemental Information 3 [file peerj-cs-06-322-s003.zip › argos3/doc/api/standalone/a00402.html]

ARGoS: core/utility/profiler/profiler.cpp File Reference


- Main Page
- Related Pages
- Namespaces
- Classes
- Files

- File List
- File Members

# core/utility/profiler/profiler.cpp File Reference

`#include "profiler.h"`  
`#include <argos3/core/utility/logging/argos_log.h>`  
`#include <argos3/core/utility/configuration/argos_configuration.h>`  

Include dependency graph for profiler.cpp:

Go to the source code of this file.

|  |  |
| --- | --- |
| Namespaces | |
| namespace | argos |

|  |  |
| --- | --- |
|  | The namespace containing all the ARGoS related code. |

| Defines | |
| #define | DUMP\_RUSAGE\_FIELD\_HUMAN\_READABLE(MSG, FIELD)   c\_os << MSG ": " << t\_resources.ru\_ ## FIELD << std::endl; |
| #define | DUMP\_RUSAGE\_FIELD\_TABLE\_ROW(FIELD)   c\_os << " " << t\_resources.ru\_ ## FIELD; |
| #define | DIFF\_RUSAGE\_FIELD(FIELD)   tResult.ru\_ ## FIELD = t\_resource1.ru\_ ## FIELD - t\_resource2.ru\_ ## FIELD; |
| Functions | |
| void | argos::DumpResourceUsageHumanReadable (std::ostream &c\_os, const ::rusage &t\_resources) |
| void | argos::DumpResourceUsageAsTableRow (std::ostream &c\_os, const ::rusage &t\_resources) |
| ::rusage | argos::operator- (const ::rusage &t\_resource1, const ::rusage &t\_resource2) |

---

## Define Documentation

|  |  |  |  |  |  |
| --- | --- | --- | --- | --- | --- |
| #define DIFF\_RUSAGE\_FIELD | ( | FIELD |  | ) | tResult.ru\_ ## FIELD = t\_resource1.ru\_ ## FIELD - t\_resource2.ru\_ ## FIELD; |

Definition at line 88 of file profiler.cpp.

|  |  |  |  |  |  |
| --- | --- | --- | --- | --- | --- |
| #define DUMP\_RUSAGE\_FIELD\_HUMAN\_READABLE | ( | MSG, |  |  |  |
|  |  | FIELD |  | ) | c\_os << MSG ": " << t\_resources.ru\_ ## FIELD << std::endl; |

Definition at line 36 of file profiler.cpp.

|  |  |  |  |  |  |
| --- | --- | --- | --- | --- | --- |
| #define DUMP\_RUSAGE\_FIELD\_TABLE\_ROW | ( | FIELD |  | ) | c\_os << " " << t\_resources.ru\_ ## FIELD; |

Definition at line 62 of file profiler.cpp.

---

Generated on 10 Jul 2018 for ARGoS by 
 1.6.1
